# Chemodynamic nanomaterials for cancer theranostics

**DOI:** 10.1186/s12951-021-00936-y

**Published:** 2021-06-28

**Authors:** Jingqi Xin, Caiting Deng, Omer Aras, Mengjiao Zhou, Chunsheng Wu, Feifei An

**Affiliations:** 1 Institute of Medical Engineering, Department of Biophysics, School of Basic Medical Science, Health Science Center, Xi’an Jiaotong University, No. 76 Yanta West Road, Xi’an, Shaanxi 710061 People’s Republic of China; 2 Department of Radiology, Memorial Sloan Kettering Cancer Center, New York, NY 10065 USA; 3 Department of Pharmacology, School of Pharmacy, Nantong University, 226000 Nantong, Jiangsu People’s Republic of China

**Keywords:** Fenton reaction, Chemodynamic therapy, Theranostics, Hypoxia, Combination therapy

## Abstract

It is of utmost urgency to achieve effective and safe anticancer treatment with the increasing mortality rate of cancer. Novel anticancer drugs and strategies need to be designed for enhanced therapeutic efficacy. Fenton- and Fenton-like reaction-based chemodynamic therapy (CDT) are new strategies to enhance anticancer efficacy due to their capacity to generate reactive oxygen species (ROS) and oxygen (O_2_). On the one hand, the generated ROS can damage the cancer cells directly. On the other hand, the generated O_2_ can relieve the hypoxic condition in the tumor microenvironment (TME) which hinders efficient photodynamic therapy, radiotherapy, etc. Therefore, CDT can be used together with many other therapeutic strategies for synergistically enhanced combination therapy. The antitumor applications of Fenton- and Fenton-like reaction-based nanomaterials will be discussed in this review, including: (iþ) producing abundant ROS in-situ to kill cancer cells directly, (ii) enhancing therapeutic efficiency indirectly by Fenton reaction-mediated combination therapy, (iii) diagnosis and monitoring of cancer therapy. These strategies exhibit the potential of CDT-based nanomaterials for efficient cancer therapy.

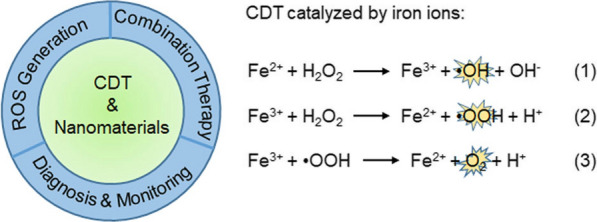

## Background

Cancer poses a serious threat to human health and has attracted considerable attention in biomedical research over the past few decades [1]. The mortality rate of cancer increases year by year, making it of utmost urgency to develop even more efficient cancer therapeutic strategies. There are many cancer therapeutic strategies, ranging from traditional strategies such as surgery, chemotherapy, radiotherapy, photodynamic therapy (PDT), to newer strategies such as photothermal therapy (PTT) and immunotherapy [2–6]. Often, the anticancer efficiency of these strategies is limited by low tumor selectivity, systemic toxicity, and adverse reactions, etc. [7]. Besides, many types of cancer gradually develop resistance to traditional therapies [8]. In order to improve the therapeutic effect, novel anticancer drugs and strategies are still desired [9, 10]. Chemodynamic therapy (CDT), which is defined as in-situ treatment via Fenton and Fenton-like reactions, has attracted more and more attention in recent years [11–13]. As a novel modality for cancer treatment, CDT exhibits preferable therapeutic performance and satisfying biosafety [14]. In comparison with traditional therapies, CDT can generate spatiotemporal controllable and tissue depth-unlimited reactive oxygen species (ROS) and oxygen (O_2_) in response to the high level of hydrogen peroxide (H_2_O_2_) in the tumor microenvironment (TME) [14–17].

Fenton and Fenton-like reactions, which generate abundant oxidative •OH or O_2_, can be triggered by the endogenous H_2_O_2_ of cancer cells and catalyzed by transition metal ions or their complexes [18]. While Fenton and Fenton-like reactions are commonly suppressed by the insufficient H_2_O_2_ and slightly alkaline conditions of a normal microenvironment, in the acidic TME, metal ions can be dissolved from iron or other transition metal ions-based nanomaterials to activate Fenton and Fenton-like reactions [19]. On the one hand, the production of •OH can result in oxidant damage to cellular constituents (proteins, lipids, DNA, et al.), apoptosis or necrosis, and likely cell death [20, 21]. The generated •OH can inhibit tumor growth directly without damaging normal tissue [22]. On the other hand, Fenton and Fenton-like reactions can generate O_2_ to relieve tumor hypoxia, which can lead to path-breaking enhanced cancer therapeutic combination strategies [23]. It is well known that tumor hypoxia is a result of an insufficient supply but quick oxygen consumption in the fast-growing neoplastic cell population in solid tumors [24, 25]; tumor hypoxia has been implicated as one of the major factors that limits treatment efficacy and that promotes development of resistance in many cancer therapeutic methods [26–28]. Fenton and Fenton-like reactions are important strategies to overcome hypoxia via in-situ production of O_2_ in the TME [29]. When used with other anticancer strategies, Fenton and Fenton-like reactions can enhance the tumor sensitivity to other anticancer strategies by supplying O_2_ to the hypoxic TME, resulting in enhanced anticancer efficacy.

**Scheme 1.**
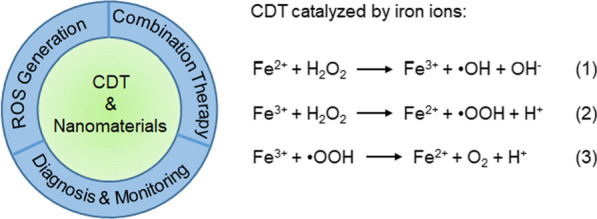
The medical applications of CDT-based nanomaterials and the representative reactions for CDT

Recently, nanotechnology has played a significant role in the development of new targeted tumor diagnosis and therapeutic strategies [30–32]. Emerging Fenton and Fenton-like reaction-based nanomaterials are mainly iron-based, followed by other metal-based nanoparticles (NPs) (including Mn^2+^, Cu^2+^, and Ti^3+^ ions, etc.), and several organic NPs [33–35]. Due to the characteristics of these nanomaterials, including tumor targeting ability, large specific surface area, and high reactivity, they are potent tools for the production of •OH and O_2_ via Fenton and Fenton-like reactions [36]. With the tumor targeting ability of these nanomaterials, Fenton- and Fenton-like reactions-based NPs can lead to gains in therapeutic efficiency while causing only a low level of side effects [11]. Herein, we present an overview of recent findings related to Fenton- and Fenton-like reaction-based nanomaterials, which can be categorized into three groups for different applications in cancer theranostics: (i) producing abundant ROS in situ to kill cancer cells directly, (ii) enhancing the therapeutic efficiency indirectly by Fenton- and Fenton-like reaction-mediated combination therapy, (iii) diagnosis and monitoring of cancer therapy (Scheme 1).

### Fenton and Fenton-like reactions for in-situ ROS generation

**Fig. 1:**
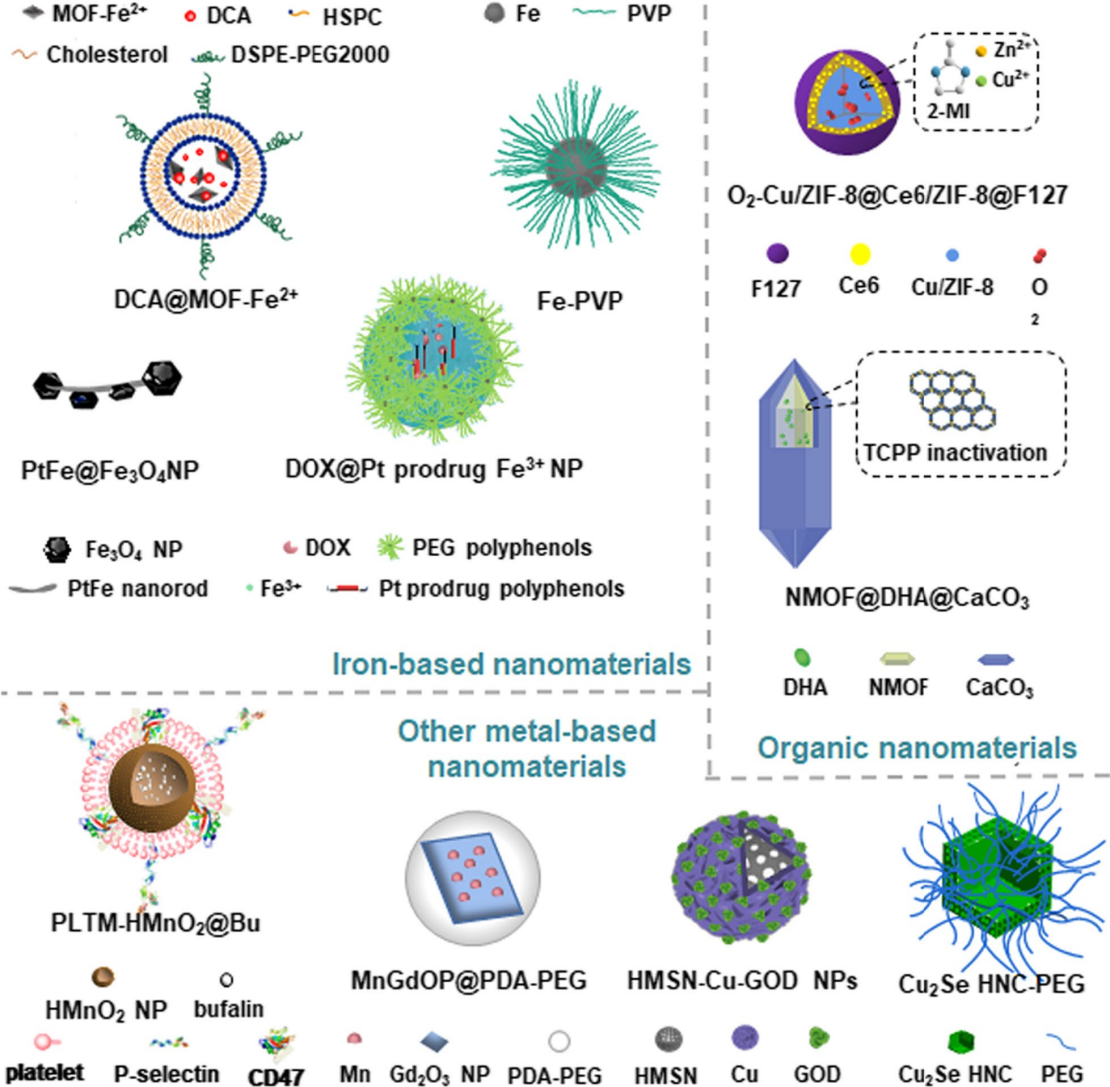
Representative nanomaterials for CDT, including iron-based nanomaterials, organic nanomaterials and other metal-based nanomaterials

Cancer cells typically already have more ROS compared with normal cells due to their increased metabolism, increased receptor signaling, and malfunction of oncogene activity and mitochondria. ROS at low levels play an important role in supporting cellular life cycles [37]. By contrast, high concentrations of ROS damage cellular constituents [38]. In particular, •OH radicals cause more serious damage to cancer cells than other ROS, which is ascribed to their relatively stronger reactivity with cellular constituents [39]. Fenton and Fenton-like reactions triggered by iron or other metal ions exhibit the ability to generate abundant •OH by decomposing the endogenous H_2_O_2_ in cancer cells [40, 41]. Therefore, Fenton and Fenton-like reactions can be used to treat cancer by in-situ production of abundant ROS to kill cancer cells directly [42], but first, the selection of the catalyst is pivotal [43]. Several nanomaterials can efficiently trigger Fenton and Fenton-like reactions, due to their high catalytic efficiency and excellent biocompatibility; these include iron-based nanomaterials, other metal-based nanomaterials (including Mn^2+^, Cu^2+^, and Mo^3+^, etc.), and several organic nanomaterials (Fig. 1) [44–47]. These nanomaterials can serve as efficient catalysts for various theranostic applications (Table 1).

**Table 1:** Representative Fenton- and Fenton-like reaction-based nanomaterials and their typical applications in cancer theranostics

Nanomaterials	Fenton nanoagents	Applications	Refs.
Iron-based nanomaterials	Dichloroacetic acid@MOF-Fe^2+^	CDT	[48]
	DOX-ferrocene-inhibitor@β-CD NPs	CDT-chemotherapy	[49]
	DOX@Pt prodrug-Fe^3+^ NPs	CDT-chemotherapy	[50]
	Fe_3_O_4_/triphenylphosphine/PpIX NPs	CDT-PDT	[51]
	PtFe@Fe_3_O_4_ NPs	CDT-PTT	[52]
	Fe-porphyrin@Bis(DPA-Zn)-RGD/(SOD2)-siRNA NPs	CDT-SDT	[19]
	Hollow Fe_3_O_4_ mesocrystals NPs	CDT-MHT	[53]
	Zero-valence iron-polyvinyl pyrrolidone NPs	CDT-MHT-PTT	[54]
	Fe-MIL-100/GOD@HA-PDA NPs	All-in-one agent	[55]
Mn-based nanomaterials	Thiol-functionalized MSN NPs@MnO_2_ NPs	CDT	[56]
	Bufalin@hollow MnO_2_ NPs	CDT-chemotherapy	[57]
	Mn-carbon dots NPs	CDT-PDT	[58]
	Mn-Gd_2_O_3_@PDA NPs	CDT-PTT	[59]
	PpIX@hollow mesoporous organosilica-MnO_x_-RGD NPs	CDT-SDT	[60]
	Hollow MnO_2_-DOX-GOD-HA NPs	All-in-one agent	[61]
Cu-based nanomaterials	Cu-TCPP MOF	CDT	[62]
	Hollow mesoporous silica-Cu-GOD NPs	CDT-chemotherapy	[63]
	Cu_2_Se hollow nanocubes-PEG	CDT-PTT	[64]
	Cu-5,10,15,20-tetrabenzoatoporphyrin nMOF and anti-PDL-1	CDT-PDT-immunotherapy	[65]
	Ce6@Cu-MOF NPs	CDT-SDT	[66]
	GOD@hollow mesoporous Cu_2_MoS_4_ NPs	All-in-one agent	[46]
Other metal-based nanomaterials	TiO_2_-ruthenium(II) complexe NPs	CDT	[67]
	MoS_2_@GOD-tirapazamine-chitosan NPs	CDT-chemotherapy	[68]
	ZIF-67-DOX@PpIX@ZIF-8@PDA NPs	CDT-PDT-chemotherapy	[69]
	WO_3-x_@γ-Poly-L-glutamic acid NPs	CDT-PTT	[70]
	TiO_1+x_-PEG NPs	CDT-SDT	[71]
	Au_2_Pt -Ce6-PEG NPs	All-in-one agent	[72]

### Iron-based nanomaterials

According to the Fenton reaction (Eq. 1 and 2), overproduction of H_2_O_2_ in the TME can be converted to •OH, •OOH and O_2_ in the presence of iron ions (Fe^2+^ and Fe^3+^) [73, 74].

1 $$ {\text{Fe}}^{{{\text{2}} + }} \, + \,{\text{H}}_{{\text{2}}} {\text{O}}_{{\text{2}}} ~ \to {\text{Fe}}^{{{\text{3}} + }} \, + \, \bullet {\text{OH}}\, + \,{\text{OH}}^{ - } $$

2 $$ {\text{Fe}}^{{{\text{3}} + }} \, + \,{\text{H}}_{{\text{2}}} {\text{O}}_{{\text{2}}}  \to ~{\text{Fe}}^{{{\text{2}} + }} \, + \, \bullet {\text{OOH}}\, + \,{\text{H}}^{ + } $$

3 $$ {\text{Fe}}^{{{\text{3}} + }} \, + \, \bullet {\text{OOH}}~ \to {\text{Fe}}^{{{\text{2}} + }} \, + \,{\text{O}}_{{\text{2}}} \, + \,{\text{H}}^{ + } $$

The Fenton reaction can be directly catalyzed by iron ions to generate •OH, •OOH, and O_2_ [75].

Meanwhile, iron ions also play a key role in ferroptosis, in which lipid-based ROS is produced due to the inhibition of system x_c_- and the biosynthesis of glutathione (GSH) [76]. In ferroptosis, the activity of system x_c_- (SLC7A11) is inhibited, resulting in decreased import of cysteine, GSH depletion, inactivation of the glutathione peroxidase 4 (GPX4), and finally ferroptosis [77, 78]. In addition, the tumor suppresser p53 can negatively regulate histone H2B monoubiquitination levels by promoting the nuclear translocation of the deubiquitinase to inhibit the expression of SLC7A11, leading to ferroptosis [79, 80]. Therefore, in addition to contributing to Fenton reaction-assisted therapy, iron ions can also produce an enhanced treatment effect via ferroptosis [81, 82].

In recent years, diverse efforts based on GSH depletion or H_2_O_2_ concentration elevation have been made to improve the anticancer efficiency triggered by Fenton and Fenton-like reactions [11, 83, 84]. GSH is overexpressed in many types of cancer cells and can reduce the ROS that are generated by Fenton- and Fenton-like reactions, thereby decreasing the therapeutic effect of CDT [85–87]. Therefore, in one study, the GSH depletion agent, sabutoclax, was loaded into Fe^3+^-containing nanoparticles (NPs) in order to produce the synergistic effect of Fe^3+^-mediated Fenton reactions and sabutoclax-induced GSH depletion [51]. In another study, the ROS inducer, β-lapachone, was loaded into the Fe_3_O_4_ NPs to produce the synergistic effect of Fe_3_O_4_-mediated Fenton reactions and β-lapachone-induced ROS generation [88]. In addition, several studies have made use of glucose oxidase (GOD), an endogenous enzyme that not only consumes GSH in cancer cells, leading to starvation therapy, but also simultaneously generates plenty of H_2_O_2_ to facilitate Fenton and Fenton-like reactions [89, 90]. For example, GOD has been loaded into an adenosine triphosphate (ATP)-responsive Fenton system (GOD@ZIF@MPN) to enhance cancer therapy [91]. The outer shell metal polyphenol network (MPN) of GOD@ZIF@MPN was degraded into Fe^3+^ and tannic acid (TA) and the internal GOD was exposed when internalized into the ATP-overexpressed tumor cells. TA reduced Fe^3+^ to Fe^2+^, and GOD reacted with endogenous glucose to generate tremendous H_2_O_2_ for Fe^2+^-catalyzed Fenton reaction. The combination of this Fe^2+^-induced Fenton reaction with ferroptosis synergistically resulted in an improved antitumor effect [92].

**Fig. 2:**
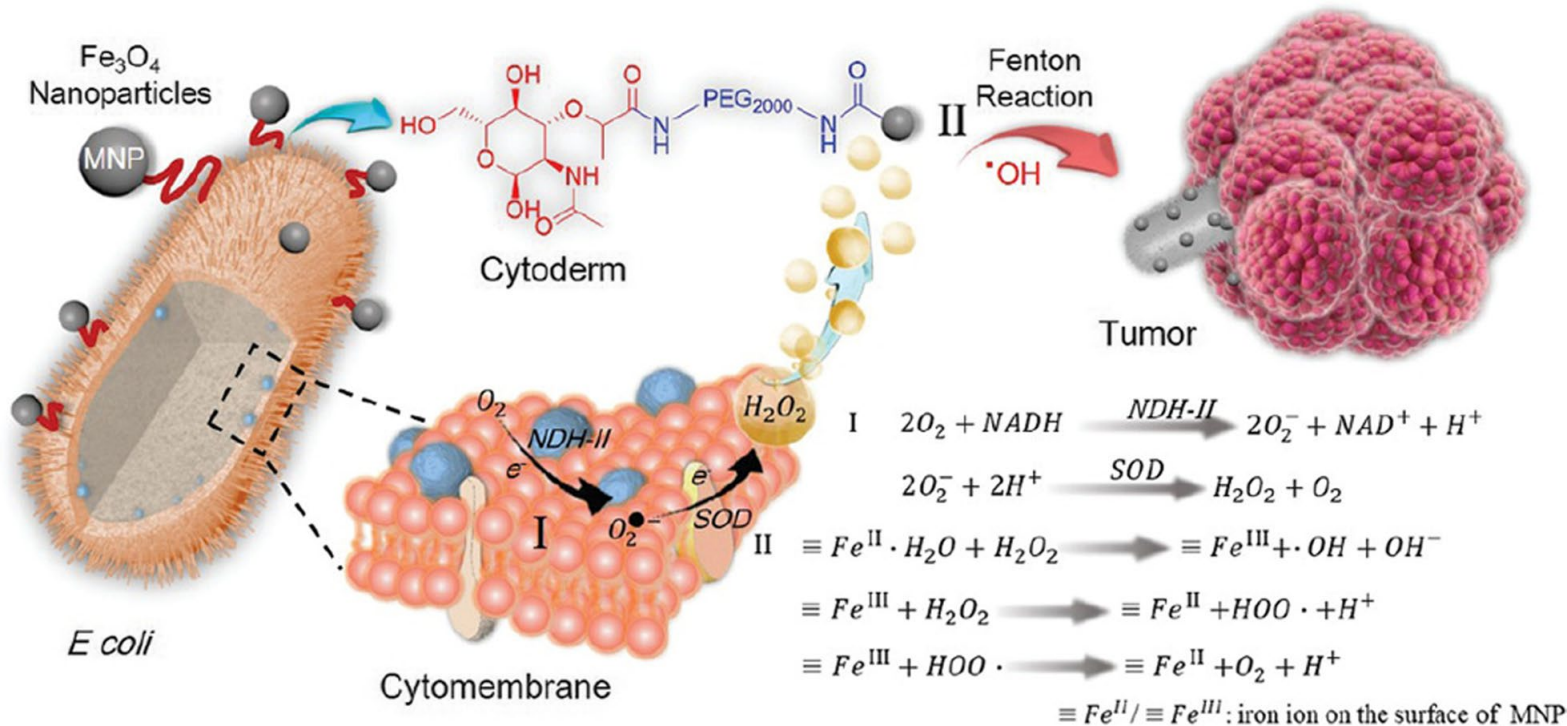
**a** The scheme of engineered bacteria as a Fenton-like reactor for tumor CDT. Reproduced with permission from Ref. [95]

In order to further improve Fenton reaction-assisted cancer therapy, one study delivered exogenous H_2_O_2_ to the tumor to increase the level of H_2_O_2_ in the TME [93]. The liquid H_2_O_2_ was loaded in the form of H_2_O_2_/Fe_3_O_4_- PLGA polymersome, in which H_2_O_2_ located in the hydrophilic core and Fe_3_O_4_ NPs inside the polymersome membrane. Upon exposure to ultrasound, the encapsulated H_2_O_2_ was released to react with the packed Fe_3_O_4_ NPs to yield •OH via the Fenton reaction, and finally improve the therapeutic efficacy. Another study used the in situ generation of H_2_O_2_ to improve Fenton reaction-assisted cancer therapy, whereby H_2_O_2_ was supplied from the reaction of CaO_2_ and H^+^ [94]. In this study, CaO_2_ was integrated into theFe_3_O_4_@HA-Cy7 NPs by preparing hybrid CaO_2_ and Fe_3_O_4_ NPs with hyaluronate acid as the stabilizer and Cy7 as a fluorescence tracer. Fe_3_O_4_-mediated Fenton reaction was enhanced by H_2_O_2_ supply from the reaction of CaO_2_ and H^+^. In yet another study, *E coli* MG1655 was engineered with NDH-2 enzyme over-expression (Ec-pE) to generate H_2_O_2_ at the tumor site [95]. The chemically decorated magnetic Fe_3_O_4_ NPs (MNP) on the surface of the engineered bacteria performed as a Fenton-like reaction catalyst to convert H_2_O_2_ to cytotoxic •OH. NDH-2 transferred accepted electrons to oxygen to produce H_2_O_2_. With the accumulation of H_2_O_2_, cancer cell death was induced by plenty of generated •OH (Fig. 2). This study shows that self-supplied therapeutics can be achieved without direct H_2_O_2_ provision.

### Other metal-based nanomaterials

Manganese-based nanomaterials can release manganese ions to catalyze Fenton-like reactions, making them widely used in cancer diagnostics and therapeutics [96–98]. Owing to the enhanced spin–spin relaxation of manganese, manganese-based nanomaterials also exhibit low toxicity, tumor targeting ability, and potential magnetic resonance (MR) imaging contrast [99]. In one study, MnO_2_-containing nanoparticles were used to deplete GSH via the reaction between MnO_2_ and the thiol groups [56]. The Mn^2+^ was released due to the reaction, which consequently triggered the Fenton-like reaction. The MnO_2_-containing NPs were able to both deliver Fenton‐like Mn^2+^ and deplete intracellular GSH. Mn^2+^ induced the generation of •OH from H_2_O_2_ via the Fenton-like reaction. Upon uptake of the NPs by cancer cells, the shell of MnO_2_ underwent the reduction by intracellular GSH to form glutathione disulfide and Mn^2+^, which enhanced CDT by GSH depletion. In another study to enhance CDT, a hybrid Fe_5_C_2_@MnO_2_ NP, GOD was loaded to generate extra H_2_O_2_ [100]. Acidic TME not only induced the decomposition of MnO_2_ nanoshell into Mn^2+^ and O_2_, but also triggered the release of GOD. Then, the released GOD not only effectively exhaust glucose in cancer cells, leading to starvation therapy, but also simultaneously generated plenty of H_2_O_2_ to accelerate the subsequent Fenton reaction to produce •OH for enhanced anticancer efficacy. As metal ions not only in the NPs but also in the metallic complex could trigger the Fenton-like reaction, in yet another study, using a Mn-Cu bimetallic complex, the Mn(II) catalyzed Fenton-like reaction to produce ROS to induce tumor cell death [101]. In addition, the copper depleted GSH to prevent ROS depletion, which further enhanced the CDT effect.

Besides manganese, copper-based nanomaterials have attracted broad attention in recent years because of their low cost, high natural abundance, and easy synthesis [102]. Particularly, copper-based nanomaterials can induce the Fenton-like reaction in wide pH range, with comparable and even better performance compared to iron-based nanomaterials [103]. It has been reported that Cu_2-x_S-PEG nanodots (≤ 5 nm) can produce high amounts of •OH by decomposing tumor-overexpressed H_2_O_2_ [104]. The generated •OH exhibited an augmented synergistic cancer therapeutic effect to the nanodots’ PDT at NIR-II wavelength range (1000–1350 nm). In another study, Gd^3+^ doped CuS NPs were shown to enable MRI guidance for CDT [105]. The Cu^2+^ of NPs reacted with GSH to generate Cu^+^, which further catalyzed the Fenton-like reaction to induce tumor death. In yet another study, self-assembled copper-amino acid mercaptide NPs (Cu-Cys NPs) were intracellularly activated by both GSH and H_2_O_2_ which were overexpressed in cancer cells [106]. The Cu-Cys NPs exhibited efficient CDT for drug-resistant breast cancer. The Cu-Cys NPs reacted with intracellular GSH to deplete GSH and reduce Cu^2+^ to Cu^+^. Subsequently, Cu^+^ triggered the Fenton-like reaction to produce cytotoxic •OH. The high concentrations of GSH and H_2_O_2_ inside cancer cells were key factors to trigger the intracellular redox reactions; therefore, the Cu-Cys NPs exhibited significantly higher toxicity to cancer cells than normal cells. Apart from these studies, in one study, as GOD can be used to produce starvation therapy, it was loaded into copper-embedded hollow mesoporous silica (HMSN-Cu) NPs [63]. Once internalized in cancer cells, GOD-catalyzed starvation therapy produced gluconic acid to accelerate the Cu^2+^ release from the collapsed HMSN-Cu framework. Cu^+^-mediated Fenton-like reaction and Cu^2+^-induced GSH depletion was promising to synergistically strengthen starvation therapy. In a very recent study, a single‐atom copper species (Cu‐HNCS) was prepared and exhibited direct catalysis ability to decompose both O_2_ and H_2_O_2_ to ROS [107]. The generated ROS oxidized intracellular biomolecules automatically, which resulted in an enhanced inhibition of tumor growth. Notably, the Fenton-like reaction showed ~ 5000 times turnover frequency of Cu species than that of Fe in Fe_3_O_4_ NPs.

Besides iron, manganese and copper, Fenton-like reactions can be catalyzed by other metal cations, such as Ti^3+^, Ir^3+^, Cr^4+^, Ce^3+^, Co^3+^, and Mo^3+^ [108–111]. For example, a ruthenium complex (abbreviated as N3) was conjugated to a TiO_2_ NP to produce TiO_2_-N3 [67]. When exposing TiO_2_-N3 to light, it produced three- and four- fold more cytotoxic •OH than TiO_2_ intrinsically. In another example, using prepared MoSe_2_/CoSe_2_@PEG NPs, near infrared (NIR) light not only triggered PTT but also induced the efficient electron–hole separation to result in efficient H_2_O_2_ generation [111]. The generated H_2_O_2_ was further decomposed into •OH via the Fenton-like reaction. The system not only supplied O_2_ to relieve the tumor hypoxia but also extra H_2_O_2_ for Fenton-like reaction. These advances demonstrate that inorganic nanomaterials can be rationally designed to manipulate therapeutic platforms based on Fenton or Fenton-like reactions for efficient antitumor therapy.

### Organic nanomaterials

Besides inorganic nanomaterials, several organic agents, such as metal–organic framework (MOF) and ferrocene, can also be used as catalysts for Fenton-like reactions [112, 113].

With their strong catalytic ability, MOF materials have been widely studied as catalysts for Fenton-like reaction. Ultrathin two-dimensional MOF of Cu-TCPP nanosheets have been reported to be promising for Fenton-like reaction-assisted cancer therapy [62]. The NPs consisted of Cu^2+^ and tetrakis(4-carboxyphenyl)porphyrin (TCPP) ligand. The TCPP ligands are peroxidized by H_2_O_2_ under acidic TME, and further reduced to ROO• in the presence of peroxidase-like Cu-TCPP nanosheets and copper ions. GSH depletion by the incorporated Cu^2+^ in the nanosheets also enhanced the therapeutic efficiency.

Ferrocene is promising as a catalyst for the Fenton reaction [114]. One study explored the feasibility of using ROS-sensitive NPs (P@P/H NPs) containing ferrocene and β-cyclodextrin inclusion complex (β-CD@Fc) to prevent tumor metastasis by intracellular ROS amplification and subsequent cascade biological reaction activation [115]. PLGA-β-CD and polyethyleneimine-Fc (PEI-Fc) were conjugated with β-CD@Fc to form an ROS-responsive amphiphilic polymer (PLGA-β-CD@PEI-Fc), which was then self-assembled into NPs (P@P NPs). Finally, heparin absorbed onto P@P NPs via charge interaction and then DOX was loaded by absorption. After the P@P/H NPs were internalized by the cancer cells, they rapidly disassembled and released DOX and Fe^2+^ in the presence of H_2_O_2_, leading to rising •OH levels, which then destroyed the mitochondrial membrane and released cytochrome c to activate the caspase apoptosis pathway, which consequently resulted in a synergistic effect of chemotherapy and CDT.

## Fenton and Fenton-like reactions-mediated combination therapy

**Fig. 3:**
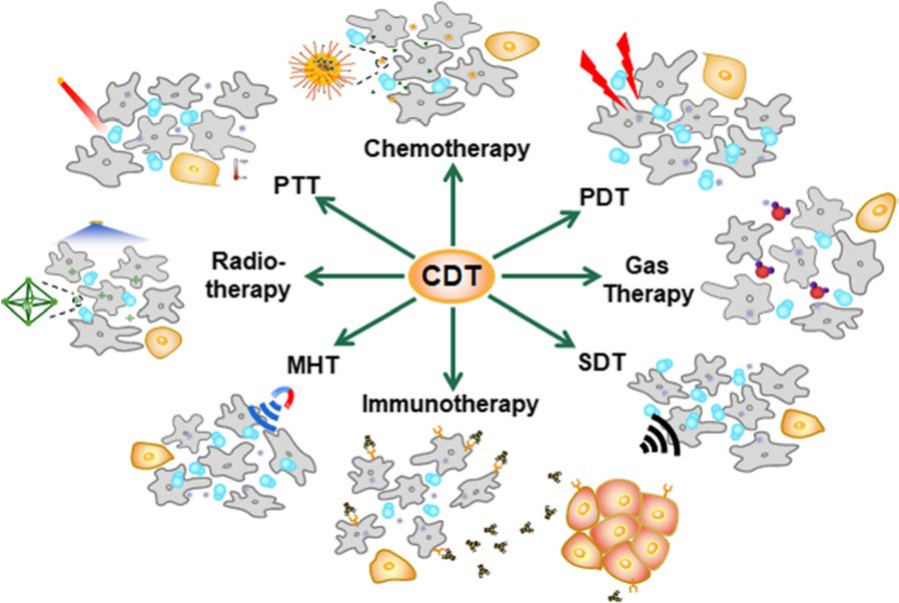
Applications of CDT in combination therapy

Combination therapy, which integrates multiple therapeutic agents into one nanoplatform, is generally more effective than monotherapy [116, 117]. For example, Vyxeos, a FDA-approved liposomal anticancer nanomedicine that is comprised of daunorubicin and cytarabine in fixed ratio, has been shown to increase the survival time of patients [118]. Nanomedicines combining Fenton/Fenton-like reactions with other therapeutic modalities may further improve anticancer efficiency. Fenton and Fenton-like reactions-mediated combination therapy can enhance therapeutic efficiency by relieving tumor hypoxia through O_2_ production or by damaging tumor cells by highly toxic ROS in situ. Therefore, multifunctional NPs for Fenton reaction-based combination therapy have increasingly been recognized as a promising strategy for tumor therapy and have been explored in combination with chemotherapy, PDT, PTT, gas therapy, sonodynamic therapy, radiotherapy, immunotherapy, and magnetic hyperthermia therapy (MHT) (Fig. 3).

### Combination of Fenton and Fenton-like reactions with chemotherapy

Chemotherapy is one of the main strategies to treat cancer. Various chemotherapy drugs, such as doxorubicin (DOX), cisplatin (CDDP), camptothecin (CPT), and tirapazamine (TPZ), have clear therapeutic mechanisms and can have remarkable therapeutic effects. However, drug resistance may develop and toxicity remains a limiting factor for chemotherapy. Recently, Fenton reaction/chemotherapy combination therapy have been demonstrated to strengthen the therapeutic efficiency of chemotherapeutic drugs with reduced side effects, even against drug-resistant cancer cells [119–121].

To enhance the therapeutic effect of loaded DOX, Fe^3+^ and Mn^2+^ have been incorporated into NPs to enable Fenton and Fenton-like reactions to generate ROS [122–124]. DOX and platinum prodrugs have also been encapsulated in Fe^3+^-polyphenol networks NPs [50], whereby the Fe^3+^-mediated Fenton reaction induced the generation of highly toxic •OH to synergize with chemotherapy. In one study, as a H_2_O_2_ inducer, β-lapachone was loaded into a pH/ROS dual-responsive NP, which consisted of pH-responsive polymer and ROS-responsive polyprodrug [125]. The NPs disassembled when triggered by low pH, and then released β-lapachone and polyprodrug. The β-lapachone supplied endogenous H_2_O_2_ for the Fenton reaction, which produced ROS to trigger DOX release, allowing combination therapy consisting of CDT and chemotherapy to take place. In another study, a ferroptosis-inducing agent was fabricated based on arginine-rich manganese silicate nanobubbles (AMSNs), which can deplete GSH efficiently and induce ferroptosis by GPX4 inactivation [126]. In addition, DOX was loaded to the surface of AMSNs to perform chemotherapy, achieving a synergistic effect of CDT and chemotherapy.

It was found that platinum (Pt) can act as ROS inducer [127]. Thus, several studies have used platinum to enhance combination therapy consisting of Fenton and Fenton-like reactions with cisplatin chemodrugs. For example, Fe_3_O_4_@PEI-Pt(IV)-PEG NPs were fabricated to enhance cisplatin-based chemotherapy by iron-mediated Fenton reaction [128]. Cisplatin activated the formation of superoxide radical (O_2_^•–^) and H_2_O_2_ from O_2_, while iron-catalyzed Fenton chemistry converted H_2_O_2_ into •OH, leading to lipid and protein oxidation and DNA damage. In another study, organic theranostic nanomedicine (PTCG NPs) were incorporated with the platinum(IV) prodrug (Pt-OH) Fe^3+^ [129]. The activated cisplatin elevated the H_2_O_2_ level, which was catalyzed by Fe^3+^ to produce cytotoxic ROS, thus enhancing the therapeutic efficacy. Apart from platinum, ferrocene has been used, whereby a ferrocene-containing nanovesicle (FcNV) was loaded with GOD and cisplatin (Pt) to combat multidrug-resistant tumors [130]. In the system, H_2_O_2_ generation was activated by Pt to enhance chemotherapy; glucose was consumed by GOD to generate H_2_O_2_ and gluconic acid for starvation therapy; and all H_2_O_2_ were catalyzed by Fenton reaction to generate cytotoxic •OH for CDT. Lastly, Mn^2+^ is also promising for combination therapy, as shown in a study where the cisplatin prodrug was anchored onto the surface of MnO_2_-coated upconversion hybrid nanocomposite, the MnO_2_ shell was degraded by intracellular GSH, and then, the released Mn^2+^ performed Fenton-like reaction for CDT to synergize with Pt-based chemotherapy [131].

**Fig. 4:**
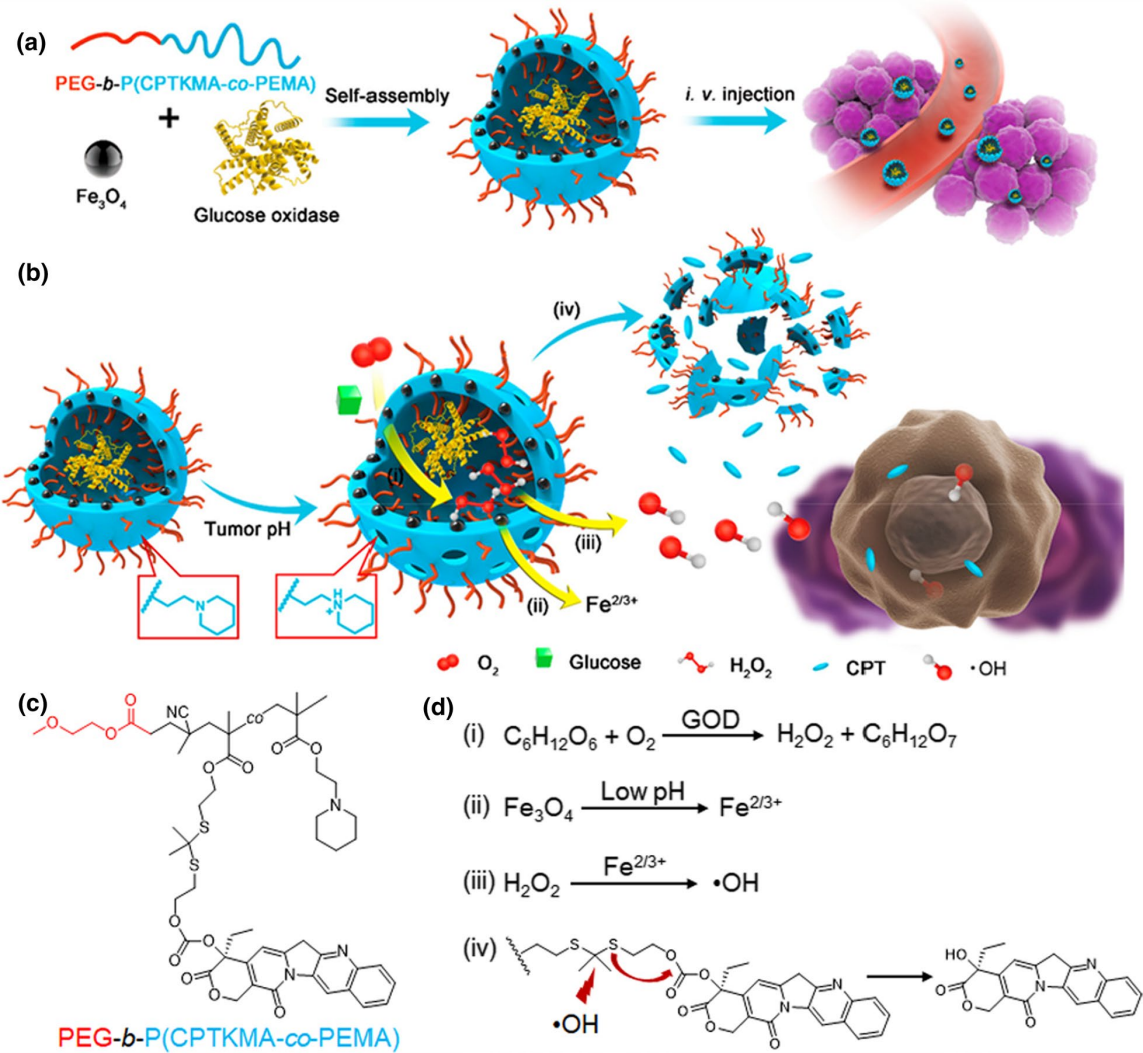
**a** Schematic illustration for nanoreactor preparation, **b** the cascade reactions in the nanoreactors triggered by tumor acidity at tumor sites, **c** the chemical structure of TME-responsive PEG-b-P(CPTKMA-co-PEMA), and **d** the cascade reactions occurring in the nanoreactors. Reproduced with permission from Ref. [134]

H_2_O_2_ performs an important role in the Fenton and Fenton-like reactions-mediated CDT; thus, extra H_2_O_2_ induced by nanomaterials is promising to enhance the cancer therapeutic effect of CPT chemodrugs. Therefore, in one study, the H_2_O_2_ inducer, β-lapachone, and camptothecin prodrug were co-loaded into iron oxide NPs [132]. The H_2_O_2_ that was induced by β-lapachone reacted with iron ions via the Fenton reaction to produce cytotoxic •OH for CDT as well as activate the release of CPT for chemotherapy. In the hybrid FeMOF NPs with Au NPs anchored on the surface and CPT loaded inside, the NPs converted glucose into H_2_O_2_ for TCPP(Fe)-mediated Fenton reaction, enhancing CDT-catalyzed chemotherapy [133]. In another study, the hybrid NPs, Fe/G@R-NRs, were prepared by loading responsive polyprodrug polymersomes with ultrasmall iron oxide NPs and GOD (Fig. 4) [134]. Triggered by the acidic TME, Fe/G@R-NRs released GOD to catalyze starvation therapy, and Fe to catalyze the Fenton reaction, which generated •OH to react with polyprodrug polymersomes to release CPT for chemotherapy.

Many other common chemotherapeutic drugs have also been explored for synergistic treatment with Fenton and Fenton-like reactions. Tirapazamine (TPZ) exhibits selective cytotoxicity for hypoxic solid tumors. Thus, in several studies, TPZ has been explored for its potential use in the Fenton reaction system, whereby oxygen was depleted and TPZ was activated [135, 136]. Furthermore, the GSH synthesis inhibitor, l-buthionine sulfoximine (BSO), has been explored to synergize with the Fenton reaction to deplete intracellular GSH, showing that this enhanced the chemotherapy and radiotherapy [137]. Mitomycin c has also been explored to synergize with the Fenton reaction by being encapsulated into superparamagnetic iron oxide NPs@PEG (SPIONs@PEG) [138]. The surface-anchored GOD catalyzed and converted glucose into gluconic acid and H_2_O_2_ for the Fenton reaction to strengthen chemotherapy. In addition, parthenolide, chloroquine, cinnamaldehyde, and bufalin have been explored as chemotherapeutic drugs to synergize with Fenton reaction-mediated CDT for an enhanced therapeutic effect [57, 139, 140].

### Combination of Fenton and Fenton-like reactions with PDT

PDT has been used for the management of neoplastic and nonmalignant diseases and is attracting more and more attention in recent years [141]. PDT is composed of three components: photosensitizers (PSs), light source, and oxygen. PSs can generate ROS under light irradiation, leading to the selective killing of tumors and cancer cells. According to the skeleton structure of their molecules, PSs are mainly divided into porphyrins, chlorins, and phthalocyanines. However, PDT has been limited by several unresolved challenges, such as inefficient therapeutic efficiency as a result of the hypoxic TME [142]. Recently, the combination of Fenton and Fenton-like reactions with PDT has been demonstrated to enhance the therapeutic antitumor effect [143, 144].

With their high ROS generation efficiency, good light stability, and low dark toxicity, porphyrins are often used as molecular probes and PSs, showing high cytotoxicity towards cancer cells under light irradiation [145]. To achieve targeted delivery to the tumor, the photosensitizer Zinc(II) protoporphyrin IX (ZnP) was inserted into the iron storage protein bacterioferritin (Bfr) in one study [146]. The inner cavity was loaded with a ferric oxyhydroxide polymer and then conjugated with polyethylene glycol on the outer surface. Under light irradiation, 3ZnP^*^ in the Bfr protein shell reduced Fe^3+^ in the enclosed [FeO(OH)]_n_ core to Fe^2+^, which subsequently triggered the Fenton reaction to generate ROS for PDT enhancement. In another study, in order to enhance the tumor accumulation of the nanomaterial, the targeted molecule AS1411 aptamer was modified onto porphyrin photosensitizer-loaded Fe_2_O_3_ NPs [147]. In yet another study, as GSH depletion plays an important role in CDT-related therapy, the hydrophobic photosensitizer 5,10,15,20-tetrakis(4-methacryloyloxyphenyl)porphyrin (TMPP) that contains multiple carbon–carbon double bonds was loaded into a nanosystem (TPFcNP) that was composed of a ferrocene-containing amphiphilic block copolymer (PEG-b-PMAEFc) [148]. The ferrocene of PEG-b-PMAEFc can catalyze H_2_O_2_ to generate •OH in the acidic TME via the Fenton reaction. The generated •OH was promising to damage cancer cells and to catalyze the addition reaction between the carbon–carbon double bonds of TMPP and the overexpressed GSH in cancer cells. The addition reaction between TMPP and GSH improved the photosensitizer hydrophilicity effectively to reduce their aggregation, leading to enhanced PDT. Increasing TME O_2_ concentration is another contribution by Fenton reaction to enhance PDT using porphyrin as a photosensitizer. In one study, a multifunctional Fenton reaction nanoplatform was fabricated [149]. The nanoplatform was prepared by co-precipitation in the presence of triphenylphosphine (TPP)-grafted dextran (Dex-TPP) and Fe^2+^/Fe^3+^ to form Fe_3_O_4_@Dex-TPP NPs. Then, the photosensitizers of protoporphyrin IX (PpIX) and glutathione-responsive mPEG-ss-COOH were modified on the surface to form Fe_3_O_4_@Dex/TPP/PpIX/ss-mPEG NPs. After internalization, Fe^2+^/Fe^3+^ were released in the acidic lysosome and then diffused into the cytoplasm, which subsequently reacted with the intracellular H_2_O_2_ to produce O_2_ and •OH for cancer therapy.

**Fig. 5:**
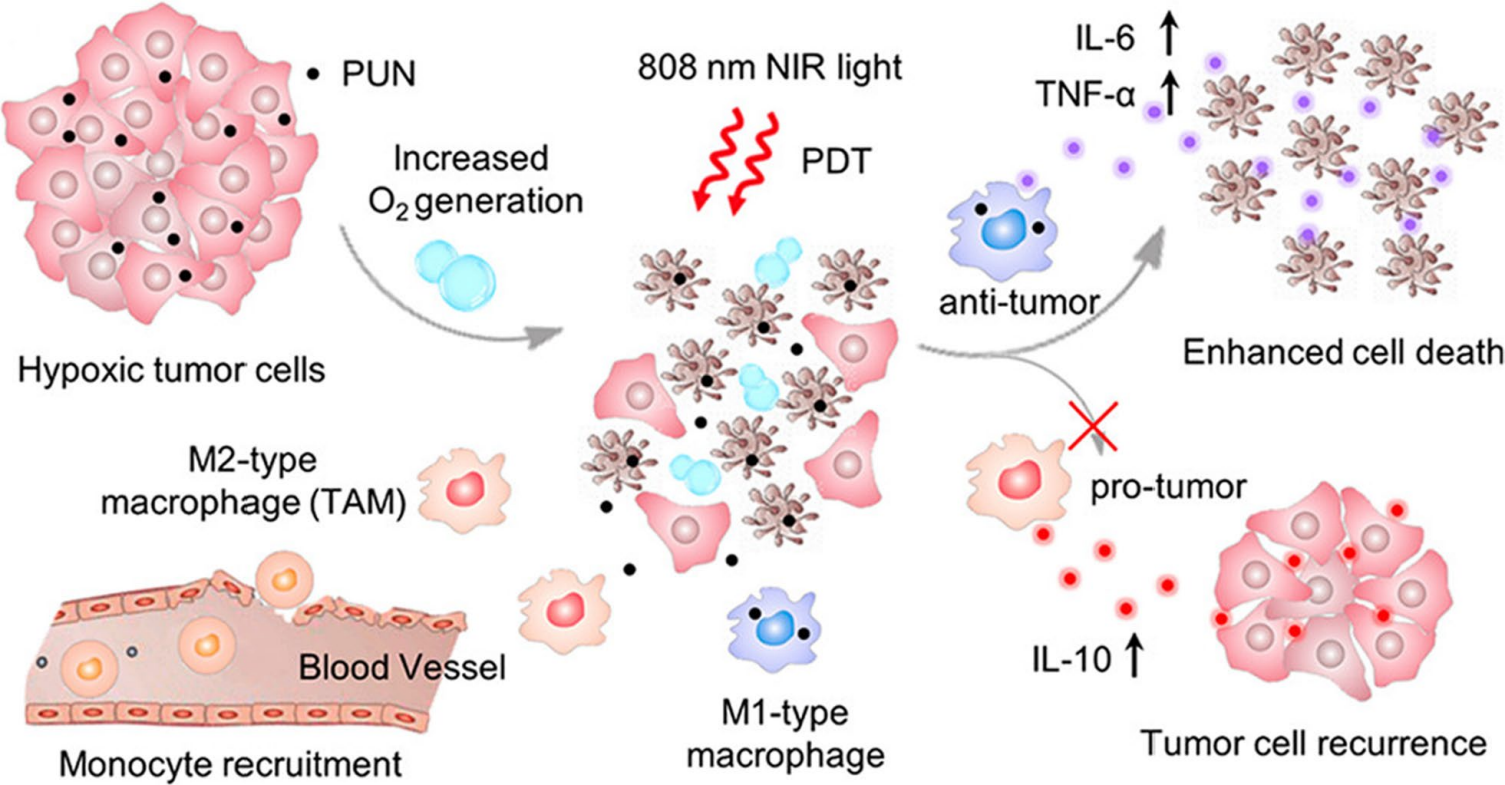
Illustration of NIR light-mediated PDT for enhanced cancer ablation in TME. Reproduced with permission from Ref. [157]

Chlorins exhibit good biological activity in antitumor, anti-pathogenic microorganisms, and anti-rheumatoid arthritis applications, which make them important candidates in the development of photosensitizers for PDT. Chlorin e6 (Ce6) is a suitable chlorin derivative for the development of PDT due to its high efficiency in producing ROS. Thus, Ce6 is a representative photosensitizer that has been explored together with Fenton and Fenton-like reactions [150]. Fenton and Fenton-like reactions decompose H_2_O_2_ into O_2_ to relieve the tumor hypoxia for enhanced PDT [151–153]. Ferrous ions not only take part in the Fenton reaction but also induce ferroptosis. Thus, in one study, the ferroptosis inducer sorafenib (SRF) was loaded into the Hb-Ce6 NPs by connecting hemoglobin (Hb) with Ce6 [154]. SRF-induced ferroptosis was shown to enhance Ce6-mediated PDT efficiently. In another study, as increased H_2_O_2_ concentration at the tumor will supply more raw material for Fenton and Fenton-like reactions to enhance PDT, the photosensitizer Cu^2+^, Au NPs, and collagenase (Col) were integrated into the framework of HMON [155]. Au NPs converted glucose into H_2_O_2_, and then Cu^2+^ catalyzed Fenton-like reaction to enhance photosensitizer mediated-PDT. In addition, Col degraded the collagen I fiber in the extracellular matrix, enhancing the NPs penetration for therapy. In another recent study, a nanoassembly was made from Ce6, carbon dots, and Cu^2+^ [156]. Cu^2+^ allowed both CDT function through Fenton-like reaction with H_2_O_2_ and GSH depletion by a redox reaction. In addition, the reduction of Cu^2+^ into Cu^+^ induced the disassembling of the NP to release Ce6, which recovered its fluorescence and ROS generation capacity. The prepared NPs acted as not only an activatable fluorescence probe but also a synergistic cancer therapeutic agent of PTT, PDT, and CDT. In yet another application of Ce6, a Ce6-loaded lanthanide-doped upconversion nanoconjugate, MnO_2_ nanosheets, and hyaluronic acid biopolymer were integrated [157]. The Fenton-like reaction produced more H_2_O_2_ in the acidic TME, and the MnO_2_ nanosheets were degraded to produce massive O_2_, enhancing the PDT effect. In addition, hyaluronic acid can reprogram the pro-tumor M2-type TAMs to anti-tumor M1-type macrophages to prevent tumor recurrence (Fig. 5).

Phthalocyanines are easy to metabolize and exhibit low phototoxicity to the skin, making them a promising alternative to porphyrins. In one study, the photosensitizer zinc phthalocyanine (ZnPc) was incorporated into the ferric pyrophosphate coordination polymer (FeP) NPs [158]. The prepared NPs released the Fe^3+^ to react with GSH, which yielded Fe^2+^ for the Fenton reaction to take place, enhancing PDT.

### Combination of Fenton and Fenton-like reactions with PTT

PTT is a type of therapy that relies on light irradiation to generate a local temperature elevation for cancer ablation [159]. The combination of Fenton and Fenton-like reactions with PTT has exhibited improved therapeutic efficiency over monotherapy [160]. Both inorganic materials and organic materials can be used as photothermal materials. Inorganic materials mainly include the noble metals (e.g., Au, Ag, and Pt), metal chalcogenides (e.g., CuS), and carbon-based nanomaterials [161, 162]. Organic photothermal materials mainly include small molecule dyes (e.g., indocyanine green, prussian blue) and conjugated polymers (e.g., polyaniline, polypyrrole, polythiophene, polydopamine) [163–165]. One main issue for PTT is that insufficient light penetration depth may lead to incomplete elimination of tumor cells, and residual tumor cells may lead to tumor recurrence and distant metastasis. The combination of PTT and Fenton/Fenton-like reactions has demonstrated great potential to achieve synergistic effects and improve therapeutic performance [166]. On the one hand, ROS production can be promoted under a higher temperature by light-enhanced Fenton reaction. On the other hand, ROS production can enhance PTT effect by sensitizing tumor cells [167].

Noble metals exhibit a strong resistance to oxidation and are regarded the most classical photothermal materials [72, 168]. Noble metals absorb light energy and then release energy in the form of heat via non-radiative decay. In addition, many metal ions released from the noble metal nanomaterials are efficient catalysts for Fenton/Fenton-like reactions [169]. In one study, Cu_2_MoS_4_/Au NPs were prepared by depositing plasmonic Au NPs into Cu_2_MoS_4_ nanosheets [170]. The prepared Cu_2_MoS_4_/Au NPs exhibited excellent photothermal conversion property and ROS generation capability, enabling synergistic therapy of PTT and PDT. Moreover, the Cu_2_MoS_4_/Au NPs converted H_2_O_2_ into O_2_ via the Fenton-like reaction to relieve tumor hypoxia and to enhance the therapeutic effect. In another study, hybrid NPs were prepared by coating upconverting NPs with iron-porphyrin MOFs on the surface and integrating Au NPs [171]. The NPs tuned the visible light harvesting ability of MOFs to excite MOFs for PDT. Moreover, the Au NPs depleted glucose and produced large amount of H_2_O_2_, resulting in enhanced Fenton reaction catalyzed by Fe^2+^ of MOFs. In yet another study, the nanozyme made of PtFe@Fe_3_O_4_ exhibited dual enzyme-like activities of both intrinsic peroxidase-like and catalase-like activities in the acidic TME [52]. On the one hand, the prepared nanozyme promoted ROS production by direct electron transfer and Fe^3+^-mediated Fenton reaction. On the other hand, ROS generation was significantly improved by the photothermal effect of nanozymes, achieving the synergistic catalytic therapy and PTT. In one last study, DOX@Fe(III)@WS_2_-PVP NPs were designed by loading Fe(III)@ WS_2_-PVP fabricated via one-pot synthesis process with DOX [172]. On the one hand, the redox reaction between Fe^3+^ and WS_2_ generated Fe^2+^ and WO_4_^2−^. On the other hand, Fe^2+^ catalyzed the Fenton reaction which generated Fe^3+^ for the redox reaction. This repetitive endogenous redox reaction resulted in biodegradation and DOX release from DOX@Fe(III)@WS_2_-PVP, leading to chemotherapy and CDT for PTT enhancement.

With their wide range of optical absorption and reasonable photothermal properties, metal chalcogenides have received great attention in recent years [173–175]. Their use has been explored to achieve synergistic combination therapy of Fenton-like activity and PTT [176, 177]. For example, in one study, (Cu_2-x_Se)-Au Janus NPs were prepared for tri-combination antitumor therapy [178]. In the designed NPs, both the Cu_2-x_Se and the Au promoted •OH generation. In addition, the Janus structure promoted photo-induced electron–hole separation to produce additional •OH. Both Cu_2-x_Se and Au contributed to PTT and promoted the reactions. As a result, synergistic tri-combination antitumor therapy was achieved. In another study, ultrasmall chalcopyrite CuFeS_2_ NPs were prepared by using bovine serum albumin (BSA) as a template [179]. The prepared NPs possessed pH-independent Cu-catalyzed Fenton-like reaction property to produce •OH for enhanced PTT. BSA was used to anchor Cu and Fe ions based on the excellent affinity of carboxyl groups and the surfactant. In yet another study, CuS NPs, another kind of NIR absorbing material for PTT was used, whereby the Cu^2+^ released from the NP was found capable to trigger the Fenton-like reaction [180]. When CuS NPs were integrated with iron-containing prodrug, the hybrid NPs not only acted as a PTT agent for photothermal ablation of tumor cells but also acted a catalyst to catalyze the regeneration of high-active Fe^2+^ from low-active Fe^3+^, enhancing Fe^2+^-mediated Fenton reaction. In another application of CuS NPs, they were integrated with MnO_2_ to generate the hybrid HMCMD NPs [181]. The HMCMD NPs possessed excellent photothermal conversion efficiency and the release of the loaded DOX was promoted under laser irradiation to enhance DOX-mediated chemotherapy. Meanwhile, Mn^2+^ released from the HMCMD NPs through a redox reaction between MnO_2_ and intracellular GSH promoted the decomposition of intracellular H_2_O_2_ by the Fenton-like reaction to generate highly toxic •OH for synergistic chemotherapy/CDT/PDT. Lastly, one study prepared CoS_2_ NPs via the self-assembly of CoS_2_ nanosclusters [182]. Co^2+^-mediated Fenton-like reaction enhanced the CoS_2_ mediated-PTT.

Carbon-based materials, such as carbon nanotubes, graphene, graphene oxide, and carbon dots, can also convert light energy to heat energy for cancer treatment [183]. Therefore, in one study, the hollow carbon matrix core, which contained ultrasmall Fe_3_O_4_ NPs, was coated with a nanoflower-like MnO_2_ shell to form a redox and light-responsive nanoplatform [184]. Fe^2+^ and Fe^3+^ released in the acidic TME catalyzed the Fenton reaction for enhanced PTT. In another study, hollow porous carbon was used to coat FeS_2_ (HPFeS_2_@C) NPs which were further loaded with tannic acid (TA) and GOD [185]. After endocytosis by cancer cells, GOD effectively catalyzed glucose to generate H_2_O_2_, which led to the generation of highly toxic •OH via Fe^2+^-mediated Fenton reaction. Meanwhile, TA reduced the generated Fe^3+^ back to Fe^2+^ to recycle the Fenton reaction for enhanced therapeutic effect.

Prussian blue is an inorganic material that contains a ferrous element and that possesses good absorption at the NIR wavelength region. Prussian blue-based nanomaterials have shown promise for both PTT and Fenton-reaction mediated CDT [186]. In one study, MIL-100 (Fe) coated K_2_Mn[Fe(CN)_6_] NPs (PBAM) were prepared for CDT and PTT combination therapy [187]. In the mildly acidic TME, the MIL-100 shell was degraded to release Fe^3+^, which further exchanged with Mn^2+^ to synthesize Prussian blue for PTT. Additionally, Mn^2+^ catalyzed the Fenton-like reaction, enhancing PTT. The in vitro data conformed that the T1-weighted MRI with Mn^2+^ contrast and photoacoustic imaging with Prussian blue contrast obtained more detailed and precise tumor information for cancer therapy. The in vivo data further exhibited that the combination of Mn^2+^-mediated CDT and Prussian blue-mediated PDT achieved synergistic anticancer performance under the guidance of PA/MRI. And with their high tumor specificity, PBAM showed promise to monitor invisible lymph node metastases and to have an accurate theranostic effect.

Ferrous-sulfur NPs are a class of nanomaterials possessing strong absorption in the NIR wavelength region, making them a good PTT agent to combine with ferrous ion-mediated Fenton reaction [188]. In one study, defect-rich Fe_3_S_4_ tetragonal nanosheets (TNSs) were fabricated by a hot-injection thermal decomposition reaction and then modified with a PVP coating [189]. PVP-coated Fe_3_S_4_ TNSs produced localized heat by PTT from the defect-rich structure, which enhanced the Fenton process by utilizing the excess H_2_O_2_ in the TME. In return, the produced •OH inhibited tumor growth and recurrence after PPT, achieving synergetic therapy of Fenton reaction and PTT. In another study, FeS_2_@C-ICG-PEG NPs were prepared by loading indocyanine green (ICG) into the FeS_2_@C yolk-shell and modifying PEG on the C yolk-shell surface [190]. FeS_2_@C-ICG-PEG possessed high photothermal conversion efficiency, enabling enhanced PTT. Besides, FeS_2_@C-ICG-PEG generated NIR-triggered ROS (•OH and O^2–^) and oxidized water to form O_2_ under NIR irradiation to relieved tumor hypoxia. Moreover, FeS_2_-mediated Fenton reaction and ICG-mediated PTT enhanced the therapeutic effect. In yet another study, thermally oxidized pyrite nanosheets (TOPY-PEG NSs) were modified on the surface [191]. The TOPY-PEG NSs catalyzed PDT via electron transfer between their FeS_2_ core and Fe_2_O_3_ shell. In addition, the Fe_2_O_3_ shell and Fe^3+^ depleted GSH and generated Fe^2+^ for the Fenton reaction, enhancing not only PDT but TOPY-PEG NSs-mediated PTT. And the fluorescent, photothermal imaging and photoacoustic capabilities of the TOPY-PEG NSs were shown to allow multimodal imaging-guided cancer treatment. In one last study, except for Ferrous-sulfur NPs, ferrous phosphide nanorods (Fe_2_P NRs) were shown to be a superior PTT agent as well as CDT agent by Fe^3+^-mediated Fenton reaction [192].

Among organic nanoplatforms, conjugated polymers with excellent optical properties have gained tremendous popularity recently. The large π-conjugated backbone and high electron delocalization structure of conjugated polymers have excellent light amplification and light harvesting properties, thus providing new opportunities in the fields of bioimaging and phototherapy [193, 194]. Polydopamine (PDA) is a kind of conjugated polymer with excellent absorption in the NIR wavelength region, which facilitates its application for PTT [195]. Thus, metal ions could be incorporated into the PDA-based NPs to enhance PDA-mediated PTT via Fenton or Fenton-like reaction-mediated CDT [196]. In order to further improve the synergistic effect of PDA-based PTT/CDT, GOD has been incorporated into the NPs to deplete glucose and supplied H_2_O_2_ for the Fenton reaction [197]. DOX has also been further loaded into such system to explore the synergistic effect of PTT/CDT/chemotherapy [198].

### Combination of Fenton reaction and sonodynamic therapy (SDT)

SDT is a cancer therapy modality triggered by low-intensity ultrasound and has emerged as a promising cancer treatment modality due to its deep penetration and non-invasive therapeutic features [199]. SDT has many advantages, including deep tissue penetration, controllability, and good patient compliance; however, its therapeutic effect is limited in the hypoxic TME and where there is low ROS release and low sensitivity [200–202]. Therefore, combining Fenton reaction with SDT can strengthen the therapeutic efficiency of either Fenton reaction or SDT [203].

Sonosensitizers like porphyrin derivatives are generally used to enhance the SDT [204]. In one study, the Fe(III)-porphyrin-based nano-sonosensitizer was fabricated by coordinating meso-tetrakis (4-sulfonatophenyl) porphyrin (TPPS) and Fe^3+^ as a core, and then anchoring with Bis(DPA-Zn)-RGD and manganese superoxide dismutase (SOD2) siRNA [205]. Fe^3+^ in the nano-sonosensitizer reduced into Fe^2+^ to deplete GSH and catalyze CDT for TPPS-SDT enhancement. In another study, another multifunctional nanosonosensitizer (PpIX@HMONs-MnOx-RGD, designated as PMR) was prepared by integrating a MnO_x_ component with hollow mesoporous organosilica NPs (HMONs), protoporphyrin (PpIX) and cyclic arginine-glycine-aspartic pentapeptide [60]. These multifunctional nanosonosensitizers enhanced ultrasound-triggered SDT by improving the tumor oxygen level. The MnO_x_ in the nanosonosensitizer acted as a nanoenzyme to convert TME overexpressed H_2_O_2_ into oxygen to facilitate SDT-triggered ROS generation and to enhance SDT efficacy. There are also some reports about the use of inorganic NPs as sonosensitizers to combine with Fenton reaction. For example, Janus Au‐MnO NPs were prepared by the self-assembly of Janus Au-MnO NPs with hydrophilic thiolated PEG (PEG-SH) and hydrophobic ROS-sensitive poly-(1,4-phenyleneacetone dimethylene thioketal) (PPADT-SH) [206]. Upon ultrasound stimulation, the prepared NPs firstly disassembled into small Janus Au-MnO NPs, and then divided into Au NPs and Mn^2+^ ions. Mn^2+^-mediated Fenton-like reaction enhanced Au-mediated SDT. In another study, PtCu_3_ nanocages were synthesized via one-pot solvothermal method, and then coated with PEG [207]. PtCu_3_-PEG nanocages acted not only a sonosensitizer under ultrasound irradiation but also a catalyst for Fenton-like reaction. Besides, the copper ion of the PtCu_3_-PEG nanocages depleted GSH, strengthening the combination therapy. In yet another study using titanium oxide NPs, the introduction of the iron element enhanced both SDT and CDT and resulted in synergistic combination therapy [208].

### Combination of Fenton reaction and immunotherapy

Cancer immunotherapy has recently gained considerable attention as a novel therapeutic option; moreover, the rational combination of immunotherapy with other therapeutic modalities presents an appealing approach to improve cancer therapy [209–212]. In recent studies, Fenton reaction-based CDT-immunotherapy not only achieved the shrinkage of in situ tumor but effectively eradicated distant tumors [213].

**Fig. 6:**
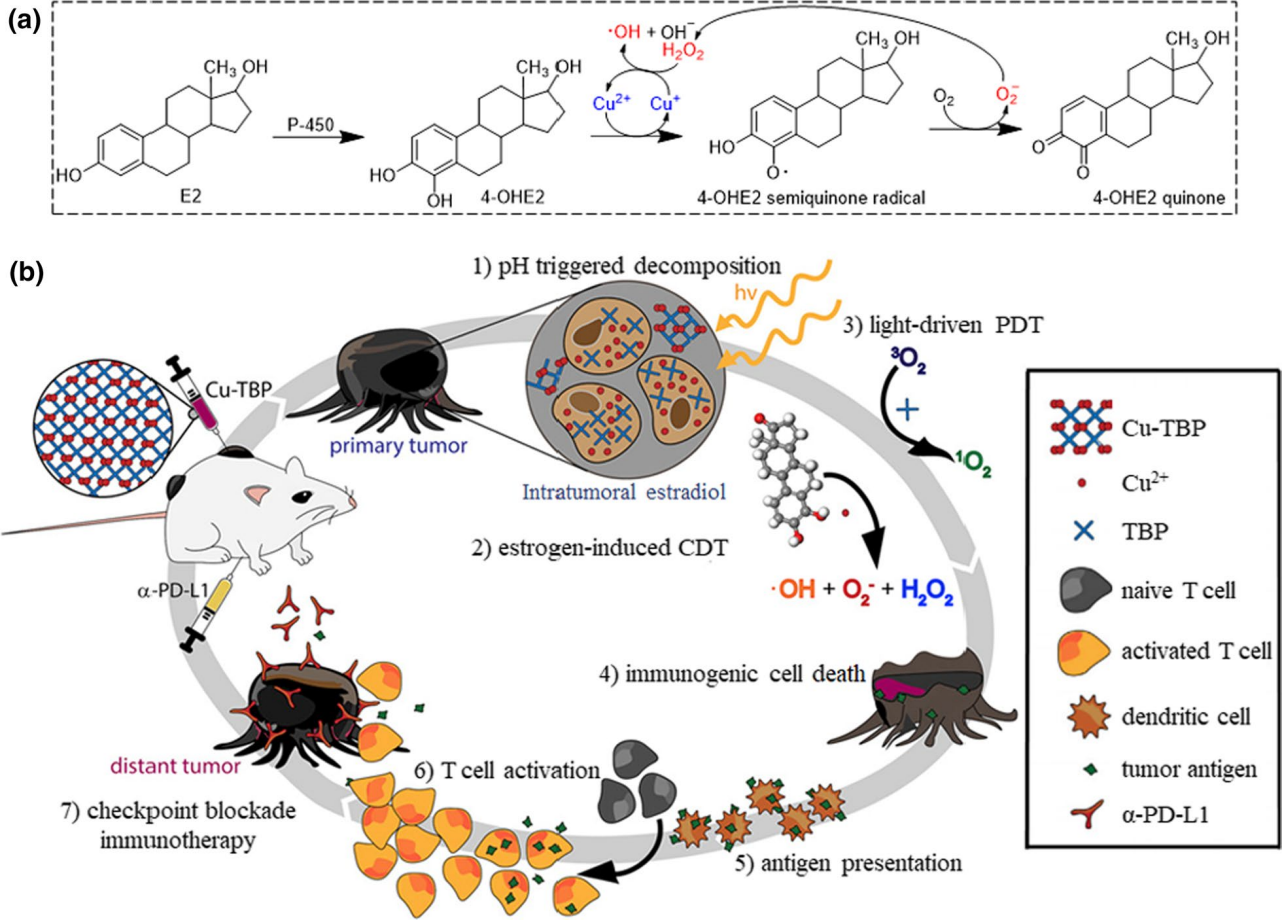
**a** Hormone-induced Cu-mediated ROS generation process. **b** Synergy of checkpoint blockade immunotherapy and nMOF-mediated radical therapy triggered by both hormone and light stimulation. Reproduced with permission from Ref. [214]

The Fenton reaction can be used to modulate cellular metabolic processes for antitumor activities, e.g., hijacking the estrogen metabolic pathway [214]. High serum levels of estrogens, especially estradiol (E2), are associated with increased cancer risks. Estrogens were found to form stable adducts with DNA by generating ROS in the downstream metabolic processes in the presence of Cu^2+^ mediated Fenton-like reaction (Fig. 6). The intratumoral administration of free Cu^2+^ can potentially hijack the estrogen metabolic pathway and trigger cytotoxic ROS generation to reduce cell-proliferation-induced tumorigenesis and enhance effective radical therapy.

It is well known that radiotherapy (RT) can trigger the immune response but its anticancer efficacy has been limited by inadequate T cell infiltration. Thus, porous Hf-based nMOFs have been explored as effective radioenhancer [65]. The combination of nMOF-mediated low-dose RT with the anti-programmed death-ligand 1 (anti-PD-L1) antibody exhibited effective therapeutic effects to distant tumors via abscopal effects. When copper was doped into hybrid NPs, the resultant product exhibited synergistic PTT, PDT, CDT as well as activated immune responses effectively [215]. Further combination with anti-PD-L1 checkpoint blockade therapy resulted in successful suppression of distant tumor growth and cancer metastasis.

It is also well known that the TGF-β inhibitor can regulate the TME to induce macrophage polarization from M2 to M1, which can regenerate H_2_O_2_. Thus, the TGF-β inhibitor has been loaded into the PEGylated iron manganese silicate NPs (IMSN) to trigger the synergistic cancer therapy [216]. The IMSN exhibited both peroxidase-like and catalase-like activities in the acidic TME, thus generating •OH and O_2_, respectively. The generated H_2_O_2_ induced by TGF-β inhibitor was an efficient supplement to the TME for IMSN-based combination therapy.

### Combination of Fenton reaction and magnetic hyperthermia therapy (MHT)

MHT is a strategy using an external alternating magnetic field to induce the relaxation and hysteresis loss of magnetic NPs, resulting in magnetic-heat conversion for cancer therapy [98]. MHT can treat tumors by producing localized heat with minimum adverse effects and without depth limitations [217]. The combination of the Fenton reaction and MHT is promising to produce more effective treatment with less side effects [54].

Hollow Fe_3_O_4_ mesocrystals have been shown to enhance Fe_3_O_4_-mediated MHT by the Fenton reaction [53]. On the one hand, the Fenton reaction produced •OH which induced cell apoptosis and reduced the expression of heat shock proteins, enabling low-temperature-mediated magnetic hyperthermia. On the other hand, in situ hyperthermia in turn facilitated the Fenton reaction to achieve synergistic combination therapy. In another study, the lipophilic iridium(III) cation in the Ir(III) complexes modified MnFe_2_O_4_ NPs acted as a target ligand to the tumor [218]. On exposure to an alternating magnetic field, the MnFe_2_O_4_@Ir NPs caused localized hyperthermia, leading to mitochondrial damage and even cell death. Moreover, Fe^3+^ on the surface of NPs converted into Fe^2+^ by reacting with GSH, catalyzing the Fenton reaction to enhance magnetic hyperthermia. In yet another study, the further loading of GOD not only resulted in starvation therapy but also generated more H_2_O_2_ at the tumor, resulting in synergistic starvation-chemodynamic-hyperthermia tumor therapy [219]. Lastly, one study showed that in graphene oxide-grafted magnetic manorings, the elaboration of ferrimagnetic vortex-domain iron oxide nanoring and graphene oxide exhibited efficient thermal conversion and amplified the Fenton reaction-induced ROS level significantly under an alternating magnetic field [220]. The magnetothermal effect and ROS generation activated the in vivo immunologic effect to carry out efficient MHT in vivo.

### Combination of Fenton reaction and RT

RT is a widely used cancer therapeutic modality in clinic. Tumor hypoxia is a major reason for RT resistance. As Fenton and Fenton-like reactions can produce O_2_ to relieve tumor hypoxia, they are promising to enhance RT [221]. Traditionally, the therapeutic efficacy of RT is a result of direct radiation damage and indirect damage caused by •OH. In one study, Janus-like Au-Fe_2_C NPs that performed as Fenton reaction catalysts were synthesized to sensitize RT by generating more hydroxyl radicals (•OH) than that by pure Au NPs under radiation [222]. The catalysis-based radiosensitization strategy exhibited improved anticancer performances both in vitro and in vivo.

To further improve the sensitization effect by the Fenton reaction, in another study, Hafnium-based nanoscale metal − organic frameworks (Hf-nMOFs) with uniformly dispersed Fe^3+^ were constructed [223]. The NPs generated tremendous ROS, resulting in persistent ROS stress and reassorted cell cycle distribution. In addition, high-energy electrons resulting from Hf^4+^ partially converted H_2_O to •OH and relaxed to a low-energy state to facilitate the reduction of Fe^3+^ to Fe^2+^ that promoted the production of •OH via the Fenton reaction. The NPs exhibited improved radiotherapeutic effects on tumors by both the Fe^2+^-based Fenton reaction and Hf^4+^-induced X-ray energy conversion. In yet another study, the incorporation of anti-PD-L1 improved the local therapeutic effect of RT to distant tumors [65].

To improve the selectivity and controllability of the combination therapy of Fenton-like reaction and RT, a TME-responsive Cu_2_(OH)PO_4_ NP was synthesized [224]. The Cu_2_(OH)PO_4_ NP was responsive to both H_2_O_2_ and X-ray. Under X-ray irradiation, the NP generated CuI sites to serve as a Fenton-like catalyst to decompose overexpressed TME H_2_O_2_ into cytotoxic •OH. Meanwhile, the Fenton-like reaction was limited within normal tissues and organs due to lower H_2_O_2_ level compared to that at the tumor. This TME responsive Fenton-like catalyst ensured the radiosentization in hypoxic tumors but not in normal cells.

### Combination of Fenton reaction and gas therapy

It is well known that some gases perform as signal molecules in vivo that influence many pathophysiological processes such as cancer. Therefore, controllable gas release and tumor-targeted gas delivery is highly desired to avoid the risk of poisoning normal tissues and organs, and to amplify their cancer therapeutic effect [225].

In a study using Fe_1−x_S NPs, the increased temperature induced by NIR light irradiation prompted Fenton reaction-mediated •OH generation [226]. In addition, these NPs produced H_2_S gas in the acidic (pH = 6.5) TME, which suppressed the enzyme activity of cytochrome c oxidase to inhibit the tumor growth. Both in vitro and in vivo results demonstrated that the H_2_S-mediated gas therapy and Fenton reaction-mediated CDT produced a synergistically enhanced antitumor performance, thereby opening up a new method for gas‐mediated cancer treatment. In another study, the released H_2_S gas produced extra suppression on the intracellular catalase activity of cancer cells to induce the accumulation of H_2_O_2_ that facilitated Fenton reaction-mediated ROS generation [227].

Nitric oxide (NO) is another signal molecule exhibiting multiple antitumor activities [228]. Therefore, one study incorporated a GSH-sensitive NO donor into the iron-based nanoscale coordination polymer (NCP) [229]. The high level of GSH in tumor cells triggered the specific release of NO in situ. Meanwhile, Fe^2+^-mediated Fenton reaction at the tumor generate cytotoxic •OH for CDT. In addition, the Haber–Weiss reaction between Fe^2+^ with H_2_O_2_ produced plenty of •O_2_, which further reacted with NO to generate ONOO^−^, which was more cytotoxic than •O_2_^−^ or NO.

Though carbon monoxide (CO) poisoning is harmful to human life, the controllable release and tumor targeted delivery are allow its use as an antitumor agent. In one study, in order to apply metal carbonyl complexes in an antitumor agent, the iron pentacarbonyl (Fe(CO)_5_) was incorporated inside an Au nanocage under an oxygen-free atmosphere [230]. The Fe(CO)_5_ formed an iron oxide shell on the nanocage surface under aerobic conditions to avoid the leakage and oxidation of the caged Fe(CO)_5_. The resulting NPs could be activated under NIR light irradiation to generate CO and iron ions, triggering CO-mediated gas therapy and iron ion-mediated CDT. The generated CO and iron ions exhibited excellent synergistically enhanced antitumor effects.

## Diagnosis and monitoring of cancer therapy

Cancer theranostics, an approach that integrates diagnosis and treatment, has been a hot research topic of late [231]. Various imaging modalities can be combined with various therapies to form tumor-targeted multifunctional nanoprobes that aim at improving the identification of malignant tumors while also having a significant treatment effect [232].

**Fig. 7:**
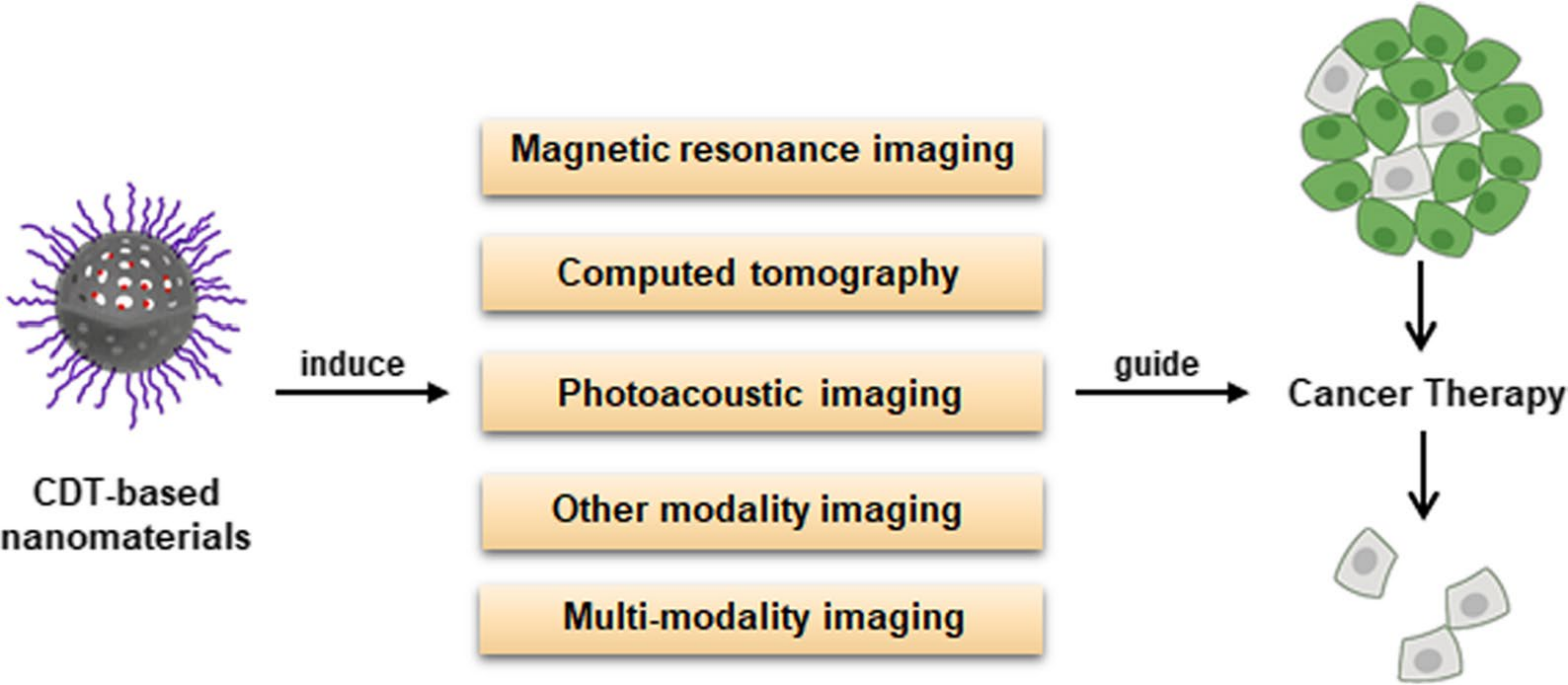
Applications of CDT-based nanomaterials for various imaging modalities

In addition to inducing the death of tumor cells, Fenton and Fenton-like reactions be applied for diagnosing cancer and monitoring treatment response to guide therapies. Towards this end, various Fenton and Fenton-like-based nanosensors have been adapted for use with different imaging modalities including magnetic resonance imaging (MRI), computed tomography (CT), and photoacoustic imaging (PAI) (Fig. 7) [233, 234].

### MRI-guided cancer theranostics

Owing to its superior sensitivity, high spatial resolution, and non-invasive and radiation-free characteristics, MRI has been widely used in clinical diagnosis [235]. In MRI, the atomic nuclear magnetization signal is reconstructed and converted into two-/three-dimensional images which are used for monitoring in vivo therapeutic performance [236]. To improve signal enhancement at the tumor site, TME-responsive NPs are beneficial [237].

The combination of MRI and cancer therapy has been demonstrated as an effective theranostic strategy. Recently, tumor-responsive PEGylated antiferromagnetic pyrite (FeS_2_) NPs were developed to generate an enhanced MRI signal [238]. The FeS_2_-PEG NPs promoted the Fenton reaction after entering the tumor cells, which not only led to the production of toxic ROS but also allowed H_2_O_2_ content in the tumor area to be monitored via T1 and T2 signal enhancement, thus enabling MRI monitoring of therapeutic performance. In addition, the localized heat generated by FeS_2_-PEG NPs accelerated the intratumoral Fenton reactivity and achieved a synergetic PTT/CDT effect. This MRI-guided synergetic nanoplatform opens up a new direction for anticancer theranostics.

In another study, to enhance the sensitivity of MRI, switchable MRI-guided core–shell-structured Fe_5_C_2_@Fe_3_O_4_ NPs with a Fe_5_C_2_ core and a Fe_3_O_4_ shell were fabricated [239]. Compared with Fe_3_O_4_ NPs, the Fe_5_C_2_@Fe_3_O_4_ NPs exhibited high magnetization and discharged ferrous ions more effectively in acidic TME. The high magnetic property of Fe_5_C_2_@Fe_3_O_4_ NPs benefited the visualization of tumor aggregation via T2-weighted MRI and magnetic targeting. Both in vitro and in vivo studies demonstrated effective ROS generation and tumor orientation, exhibiting enhanced therapeutic efficacy and low toxicity. Besides, the ionization of Fe_5_C_2_@Fe_3_O_4_ NPs in the acidic TME reduced the T2 signal, while the ferrous ions release increased the T1 signal in MRI, which provided enhanced MRI-guided cancer therapy for tumor treatment. The Fe_5_C_2_@Fe_3_O_4_ NPs provided an effective strategy for specific cancer therapy guided further by MRI.

To overcome the biological barriers of drug delivery, in one study, a precise tumor-targeting nanozyme (Ag_2_S@Fe_2_C-DSPE-PEG-iRGD) was rationally prepared for enhanced cancer therapy [240]. This nanozyme displayed efficient intracellular uptake, intense fluorescence, and high level of ROS production in cancer cells. Moreover, this nanozyme realized high-resolution tumor monitoring in tumor-bearing mice, including superior fluorescence imaging in NIR-II and high-sensitivity MRI, which was beneficial for tumor vascular normalization. Besides, the antitumor effect of Ag_2_S@Fe_2_C-DSPE-PEG-iRGD was enhanced in vivo when combined with bevacizumab-induced tumor vascular normalization. In addition, a high-degree of Ag_2_S@Fe_2_C-DSPE-PEG-iRGD (~ 90%) could be rapidly excreted from the mice within 14 days, promoting the clinical application of the nanozyme. Hence, the combination of tumor vascular normalization and imaging-guided nanozymes may accelerate the development and clinical applications of nanomedicines.

### CT-guided cancer theranostics

Owing to its high efficiency and high resolution as well as its low cost, CT has been a widely used non-invasive clinical imaging technique [241]. Although CT exhibits a much higher resolution than several other imaging strategies, it remains difficult to distinguish small changes in tissue over time. In order to enhance the accuracy of disease diagnosis, various CT contrast agents have been designed and developed. The commonly used CT contrast agents in clinic are small molecule iodides; however, the toxicity and short circulation time limit their further application [242]. The development of nanosized contrast agents can effectively improve the diagnostic efficacy and reduce the side effects of contrast agents. Besides iodine-containing NPs, some heavy metal-based NPs (gold, bismuth, tantalum and lanthanide, etc.) are promising as CT contrast agents, exhibiting obvious advantages for the visualization imaging of blood vessels and tumors [243].

Because of their high-Z atoms, Au NPs with good biocompatibility and simple synthesis strategy are widely used as a contract agent to enhance CT imaging [244]. To develop a versatile nanoplatform for tumor theranostics, in one study, a novel Au@Prussian blue (Au@PB) nanocubes (NCs) was constructed via the templates method, and then DOX was subsequently encapsulated into the Au@PB NCs [245]. The in vitro and in vivo experiments demonstrated that the DOX-Au@PB NCs exhibited synergistic effects on tumor growth inhibition. Besides, owing to the excellent X-ray attenuation coefficient of Au, the Au@PB NCs displayed higher CT imaging efficiency (∼27.13 HU·mL·mg^–1^) than the reported CT agents. This study provides a potential strategy for CT-guided tumor therapy.

In another study, a nanocomposite (Fe_3_O_4_@PDA@BSA-Bi_2_S_3_) that was composed of Fe_3_O_4_, polydopamine, bovine serum albumin and Bi_2_S_3_ was designed for MRI/CT monitored CDT/PTT combination therapy [173]. In this formulation, the stability and biocompatibility of the NPs were rendered by BSA coating. Meanwhile, the Fe_3_O_4_ NPs not only triggered Fenton reactions and produced highly cytotoxic •OH for tumor therapy, but also acted as the MRI contrast agent for precise cancer diagnosis. Besides, the Bi_2_S_3_ component exhibited outstanding photothermal transducing ability and CT imaging capacity. Besides, the high T2-relaxation time of Fe_3_O_4_ improved the MRI contrast, while the high X-ray attenuation coefficient of Bi enhanced the CT contrast, thereby facilitating the precise monitoring and synergistic tumor therapy. This work displays a feasible strategy to construct a composite nanoplatform for tumor theranostics.

### PAI-guided cancer theranostics

Owing to its outstanding fine spatial resolution and deep tissue penetration, PAI is a promising imaging modality in clinical cancer diagnosis [246]. In PAI, the pulsed laser radiation energy is absorbed by the contrast agent and converted into an acoustic signal, and then the signal is measured and images are constructed by a scanning transducer [247]. The development of nanomaterials as PAI contrast agents with high sensitivities and optimal NIR absorption is crucial to PAI applications.

By decorating the 2,2′-azino-bis (3-ethylbenzothiazoline-6-sulfonic acid) (ABTS) on the surface of graphene quantum dot nanozyme (GQDzyme) and then camouflaging with FA conjugated natural erythrocyte membranes, a novel exosome-like GQDzyme/ABTS vesicle was developed as an in vivo H_2_O_2_-sensitive PAI agent in the treatment of nasopharyngeal carcinoma in one study [248]. In the presence of H_2_O_2_, ABTS was effectively oxidized by the peroxidase-like activity of GQDzyme. The achieved oxidized ABTS displayed strong NIR absorbance, and the GQDzyme/ABTS NP showed to be a promising catalytic PAI contrast agent with high sensitivity to H_2_O_2_ in the cancer cells. Attractively, the lysosomal escape of the nanozyme vesicles promoted their interactions with H_2_O_2_ in cancer cells. Due to the photothermal properties and optical absorption of ABTS, PA signal was selectively triggered in the cancer cells with overexpressed H_2_O_2_ based on the peroxidase-like activity of GQDzyme. In addition, GQDzyme acted as nanocarriers for delivery and tracking drugs, exhibiting optimal stealth ability and biocompatibility for long blood circulation time. Overall, the GQDzyme/ABTS-based nanozyme vesicle was shown to be a promising PAI contrast agent for non-invasive tumor imaging.

In another study, to effectively exterminate tumors, biocompatible ferrous phosphide nanorods (FP NRs) was constructed under the NIR-II laser illumination, exhibiting high photothermal conversion efficiency [249]. Owing to their excellent photothermal conversion efficiency (56.6%) and traverse relaxivity (277.79 mm^−1^ s^−1^), the FP NRs acted as optimal PAI and MRI agents, achieving dual-mode imaging guided enhanced combination therapeutic efficacy of CDT and PTT. The in vitro and in vivo experiments exhibited that the FR NRs could efficiently induce the death of cancer cells and completely exterminate tumor. The metallic phosphide-based NRs could realize deep tissue penetration under NIR-II laser irradiation and ultrasound stimuli for enhancing the antitumor effect via PAI/MRI monitoring, which provides an ideal strategy for developing multifunctional Fenton nanoplatform in future clinical applications.

### Other imaging modality-guided cancer theranostics

Surface-enhanced Raman scattering (SERS) can provide molecular fingerprint information in various cells and tissues [250]. SERS possesses remarkable advantages, including being non-invasive and having ultrahigh specificity and resolution, and resistance to autofluorescence and photobleaching [251]. In an interesting study, para-aminothiophenol (PATP) and hemin were decorated on gold NP (Au@PATP/Hemin) to detect •OH, O^2·−^, ROO·, and ^1^O_2_ directly, and H_2_O_2_ indirectly [252]. H_2_O_2_ was converted into •OH via hemin-catalyzed Fenton reaction, and PATP reacted with •OH, O^2·−^, ROO· or ^1^O_2_ by a radical oxidant coupling reaction to form 4,4’-dimercaptoazobenzene (DMAB), eliciting new Raman peaks for quantitative detection. The nanoprobes were sensitive and quantitative, showing promise to monitor tumor development and inflammation progression, and guide therapies.

The capture and inactivation of circulating tumor cells (CTCs) in blood vessels have been regarded as a promising strategy to inhibit tumor metastasis [253]. Thus, in one study, an all-in-one nanoplatform was prepared as a signal probe and CDT agent by integrating magnetic MOF (magMOF) NPs with TiO_2_ nanotube arrays (TiNTs) [254]. The magMOF NPs were constructed by encapsulating Fe_3_O_4_ with a MIL-100(Fe) shell, and then the GOD was loaded into the magMOF NPs. The gluconic acid generated by GOD enhanced TME acidity, therefore prompting the dissolution of MIL-100(Fe) to produce Fe^3+^, and then effectively improved the cell killing efficiency via the Fenton-like reaction. Attractively, the CTCs were magnetically captured and collected onto TiNTs. TiNT photocatalysis activated the release of Fe^3+^ and produced exogenous •OH radicals, which could be measured by pulse voltammetry and TiNTs as the electrode. The intensities of differential pulse voltammetry were correlated with CTC numbers ranging from 2 to 5000 cell mL^−1^. This material provides a novel method for constructing multifunctional chips for CTC capture and early tumor diagnosis.

### Multi-modality imaging-guided cancer theranostics

Precisely locating the tumor site and achieving whole-body visualization based on multimodal imaging have garnered extensive research attention for accurate and efficient antitumor therapy [255]. In one investigation, pure phase bismuth ferrite nanocatalysts (BFO NCs) were prepared for multimodal imaging-guided Fenton effect against malignant tumors [256]. The prepared BFO NCs showed excellent physiological stability and biocompatibility in the blood circulation. Interestingly, the external ultrasound assistance enhanced the ROS generation of BFO NCs, owing to the partial grievous turbulence of cavitation bubbles triggered by ultrasound, thereby promoting the transfer efficacy of the Fenton reagents. Meanwhile, the BFO NCs exhibited superior fluorescence intensity within the NIR-II laser. Besides, the high X-ray attenuation coefficient (5.74 cm^2^/kg at 100 kV) and intrinsic magnetic performance of Bi rendered BFO NCs an excellent contrast agent for CT imaging and MRI. According to the in vitro and in vivo results, the BFO NCs effectively inhibited tumor growth under the external ultrasound assistance, which was precisely monitored by fluorescence imaging, CT, and MRI. Overall, the BFO NCs presented multimodality imaging capacity and enhanced Fenton therapeutic effect, offering a promising prospect for precise and non-invasive antitumor therapy.

In the recent years, lanthanide-based downconversion NPs (DCNPs) have attracted attention because of their unique characteristics, including high stability, long luminescence lifetimes, low photobleaching, and precisely controlled size [257]. To achieve high-resolution real-time tumor observation and whole-body visualization, biodegradable copper/manganese silicate nanosphere-coated lanthanide-doped NPs (LDNPs@CMCNs) were developed for trimodal imaging (NIR-II, MRI, and CT) guided CDT/PDT combination therapy in one study [258]. The Yb^3+^/Er^3+^/Tm^3+^ were doped in the core and Yb^3+^/Ce^3+^ in the shell, enabling the ultraefficient upconversion and downconversion emissions of LDNPs upon NIR light excitation. In the hypoxic tumor, the GSH-responsive CMSNs were degraded and released Mn^2+^ and Cu^+^ ions for Fenton-like •OH generation. The Ce^3+^ and Er^3+^ were doped in the shell and core of LDNPs, respectively, allowing NIR-II imaging. In addition, lanthanide-doped DCNPs allowed T1 MRI and CT imaging. The TME-responsive release of Mn^2+^, together with self-amplified trimodal bioimaging (NIR-II luminescence imaging, MRI, and CT) makes this approach greatly promising for highly efficient tumor theranostics.

Lastly, in one study, using biodegradable manganese carbonate NPs, the release of Mn^2+^ and CO_2_ gas can be triggered by acidic TME and the released Mn^2+^ and CO_2_ can allow for MR and ultrasound imaging of tumors, respectively [259].

## Conclusions and outlook

In recent years, many studies have described innovative strategies to treat cancer on the basis of Fenton and Fenton-like reactions. This review summarizes the latest developments in Fenton and Fenton-like reactions by analyzing the purpose and mechanism of Fenton and Fenton-like reaction-based nanomaterials. Fenton and Fenton-like reactions can adequately exert their potential when combined with other therapeutic strategies, providing specific, efficient, and safe protocols for cancer treatment. Fenton and Fenton-like reaction-based nanomaterials can also be used for accurate tumor diagnosis and for monitoring tumor growth and guiding the therapy of cancers in the clinic using different imaging modalities such as MRI, CT, and PAI.

There has been significant progress in the development of Fenton and Fenton-like reaction-based nanomaterials; however, there are some problems which need to be solved in order for them to be used clinically. (i) The stability, biocompatibility, and biosafety of Fenton and Fenton-like agents need to be improved. Ultrasmall or biodegradable CDT-based nanomaterials which can be removed from the body and which exhibit a rapid metabolism can achieve better clinical safety. And relative to inorganic nanomaterials, organic molecules are more degradable and some of them also exhibit the potential of triggering Fenton and Fenton-like reactions. Therefore, the exploration of organic molecules with properties of Fenton and Fenton-like reactions may provide extra opportunities to discover biocompatible Fenton and Fenton-like agents. (ii) The traditionally understood mechanism of the Fenton and Fenton-like reactions may not adequately explain their reactions in a complicated physiological system. More attention should be paid to monitor in situ Fenton reactions and Fenton-like reactions process via on-line characterization techniques, thereby optimizing their antitumor efficacy and enhancing their clinical applications. (iii) Fenton and Fenton-like reactions can generate O_2_ to alleviate the tumor hypoxia, which is a main restriction in clinical radiotherapy. However, the exploration of their combination with other therapeutic strategies is still lacking. In addition, in improve therapeutic efficacy, CDT-based combination therapy with genetic or molecular methodologies should also be explored. (iv) The TME responsive agents of Fenton and Fenton-like reactions have exhibited improved specificity, efficacy, and safety for tumor therapy. However, there remains an urgent need to design personalized TME responsive agents for different tumors microenvironments, which will benefit their clinical translation.

Although Fenton and Fenton-like reactions-mediated synergistic strategies for cancer therapy face several challenges, the clinical translation of Fenton and Fenton-like reactions-based nanomedicines are still promising, especially after their current drawbacks have been addressed in the future.
